# Development and validation of an open data model for pharmacogenetics to enable semantic interoperability in clinical practice

**DOI:** 10.1038/s41397-026-00418-0

**Published:** 2026-06-13

**Authors:** Videha Sharma, John H. McDermott, Jessica Keen, Heidi Koikkalainen, Ian McNicoll, Angela Davies, William G. Newman

**Affiliations:** 1 The Division of Evolution, Infection and Genomics, School of Biological Sciences, University of Manchester, Manchester, M13 9PT UK; 2 Manchester Centre for Genomic Medicine, St Mary’s Hospital, Manchester University NHS Foundation Trust, Manchester, Greater Manchester M13 9WL UK; 3 freshEHR Clinical Informatics, Northamptonshire, NN15 7RP Dartford, UK; 4 Institute of Health Informatics, University College, London, London, NW1 2DA UK; 5 Centre for Computation Biology and Health, Division of Informatics, Imaging and Data Science, University of Manchester Faculty of Biology, Medicine and Health, Manchester, Greater Manchester M13 9PT UK

**Keywords:** Pharmacogenomics, Health policy

## Abstract

Pharmacogenetics uses genetic testing to improve the safety and effectiveness of prescribed medicines, yet implementation at scale remains limited due to the absence of interoperable health IT solutions that integrate results into prescribing workflows. This study aimed to develop and validate open data standards for pharmacogenetic results to enable interoperability across healthcare systems. A baseline data model was constructed using the open standard openEHR by synthesising literature, genomic sequencing outputs, and international data specifications, and refined through iterative workshops with the Global Alliance for Genomics and Health. The model underwent two rounds of structured peer review involving 24 experts from 10 countries. Mapping to HL7 FHIR was evaluated using both manual and automated approaches, including the FHIR-Connect tool. The resulting standardised pharmacogenetic data model separates test results from therapeutic implications and incorporates recognised terminologies such as SNOMED CT and HGNC. It achieved international consensus and is published on the openEHR Clinical Knowledge Manager platform. Mapping to HL7 FHIR demonstrated bidirectional information flow within healthcare systems, with automated mapping enabling scalable and reusable transformations. This work provides a framework for storing and exchanging pharmacogenetic test results, supporting semantic harmonisation, interoperability, and integration with clinical decision support systems. Open data standards for pharmacogenetic test results therefore offer a foundation for scalable implementation of pharmacogenetics in routine clinical practice.

## Introduction

Genetic testing can improve the safety and effectiveness of commonly prescribed medicines. The science describing the relationship between medicines and genetics is known as ‘pharmacogenetics’ and falls under the umbrella of precision or personalised medicine [1]. However, despite compelling evidence of clinical and economic benefit, implementation remains limited [2]. A key barrier is the lack of information technology solutions that integrate pharmacogenetic data into healthcare systems to support everyday clinical decisions [3]. Historically, terminology in pharmacogenetics has focussed on laboratory processes rather than clinical application, but clinical implementation is now a strategic priority for many healthcare systems, with the UK Government highlighting the expanding role of genomics in preventative care in the recently published NHS 10-year plan [4].

Traditionally, the output of genetic testing in areas such as rare disease or cancer is ‘genetic report’, usually a paper or PDF document that is not machine readable and often difficult to interpret. This approach has also been applied to pharmacogenetics, even though its workflows and end-users differ. Pharmacogenetics is relevant not solely in specialist care but rather in the multitude of settings where medicines prescribing routinely occurs, including general practice. Yet genetic data is often reported using complex nomenclature with alphanumeric codes that mean little to most clinicians, e.g. “*CYP2C19 Intermediate Metaboliser”* [5]. Furthermore, on its own, such data has little clinical meaning; it must be combined with the therapeutic implications for the medicine being prescribed to generate useful information that can support decision-making. There is a need to simplify genomic data for clinicians who may have limited experience of using such information [6].

Complex pharmacogenetic data must therefore be translated into clinically relevant ‘test results’ that can be understood by generalists. These results should be transferred from laboratories to clinical systems, such as Electronic Health Records (EHRs), so they are available at the point of prescribing [7]. Fundamentally, there is a need to move from lengthy specialist reports to concise test results, akin to other diagnostics. As genetic information has lifelong relevance, and prescribing is universal across healthcare, point-to-point messaging between systems is not feasible; a strategy that is ‘interoperable by design’ is required [8].

Interoperability is consistently cited as a key enabler of care coordination, patient experience and outcomes [9, 10]. Yet healthcare systems globally continue to struggle to exchange data across geographies, providers and services [11]. Moving towards open standards-based platforms and embedding interoperability into new technology initiatives could help address this [12].

Effective interoperability requires alignment of clinical concepts, sometimes referred to as semantic harmonisation [13]. The open data standard openEHR is widely recognised and supported by free tooling and an international community of modellers for structured healthcare data. Fast Healthcare Interoperability Resources (FHIR) is increasingly adopted to create standard data messages between systems. Combining these approaches can support an interoperable strategy.

Central to such a strategy is storing pharmacogenetic test results in a vendor-neutral, standardised format that acts as a single ‘source of truth’ for downstream use, including clinical decision support (CDS) and Health Information Exchange (HIE). Existing implementations have largely been confined to individual centres where data has been integrated within a single EHR. However, no regional or national implementations exist. The lack of widely adopted open data standards remains a key reason why scale-up has been limited [14]. This project aims to address this by developing open data standards using openEHR, incorporating terminologies such as SNOMED CT and other genomic ontologies to enable structured pharmacogenetic test results and support precision medicine in routine care.

## Methods

### Context

This project was supported by The University of Manchester, the NHS England Network of Excellence in Pharmacogenetics, and the Global Alliance for Genomics and Health (GA4GH). It was delivered by a multidisciplinary team across genomics, clinical medicine and health informatics.

The work formed part of the wider “Pharmacogenetics Roll Out: Gauging Response to Service” (PROGRESS, ISRCTN15390784) initiative, a pilot programme exploring barriers to pharmacogenetic testing in NHS primary care. This standards development contributed to its informatics strategy, alongside user research, service design and CDS development.

Due to mature tooling and an active community, openEHR was selected as the starting point. OpenEHR is an open standard for modelling, storing and retrieving health data [15]. Development of openEHR data models (archetypes) depends on collaboration between technologists and clinicians [16]. The Clinical Knowledge Manager (CKM) tool provides a free platform for archetype design and asynchronous Delphi-inspired peer review. CKM also serves as a library of peer-reviewed archetypes suitable for real-world use, supported by open, community-based governance.

### Data model development and consensus agreement

We developed a baseline model from existing research and refined it with input from an international community of scientific, clinical and informatics experts. The baseline model included concept headings, data elements, descriptions and their hierarchy. An expert editorial board with pharmacogenetics specialists (authors JM, JK, WN) and clinical informaticians (authors VS, IM) led the process, supported by experienced openEHR and genomic modelling experts.

We reviewed genomic sequencing outputs to identify reported data elements, mapped these in a mind map, and compared them with a previous FHIR pharmacogenetic reporting specification (2022). The mind map and background were presented at GA4GH workshops, and feedback informed the iterative design of a baseline model using the free Archetype Designer tool.

**Fig. 1:**
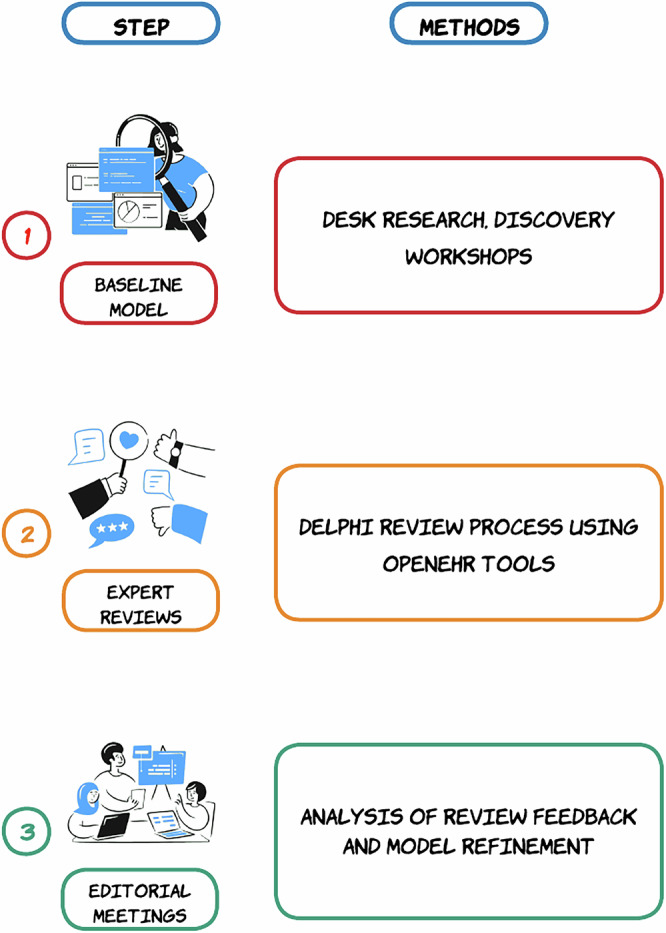
Diagrammatic overview of the data model development, international peer review process and model refinement for publication.

The model underwent two rounds of Delphi-inspired peer review with 14 and 16 reviewers, respectively, from the international openEHR and GA4GH communities. Feedback was discussed across three editorial meetings, and revisions finalised the model for publication. Figure 1 summarises the development process.

### Validation and usability testing

To validate the practical utility and alignment of the developed data model in a healthcare system, such as the NHS, we conducted semi-structured interviews with key stakeholders. This included three IT experts at NHS England’s Transformation Directorate, one national medical director, two general practitioners (GPs) and two bioinformaticians. The aim of these discussions was to gather data on challenges and opportunities to drive the adoption of the new standard. In addition, by engaging with national and front-line stakeholders we raised awareness of pharmacogenetics and the potential of precision medicine. Interviews were conducted virtually and included a short presentation to share background on pharmacogenetics, the proposed data model, and its intended use within NHS digital systems.

To ensure consistency across discussions, we used a structured topic guide covering three key themes: (1) integration into NHS systems, (2) usability and clinical decision-making, and (3) implementation and adoption. Participants were asked about the technical, clinical, and governance considerations required for successful implementation, as well as potential barriers and enablers. The semi-structured format allowed for open discussion while ensuring core topics were addressed. Feedback from these conversations was analysed to identify priority areas for implementation planning.

## Results

### Baseline data model

We identified that previous research and FHIR specifications had combined two distinct types of pharmacogenetic information within a single data model: (1) the underlying genetic test result, which describes a patient’s genetic profile and their predicted ability to process or respond to medicines, and (2) the therapeutic implications, which translate these results into prescribing guidance for specific drugs. This conflation risked limiting flexibility and reusability, as genetic results are permanent and universal, whereas therapeutic implications can vary over time with evolving evidence, drug availability, and clinical context. We hypothesised that these are distinct concepts, which should be modelled separately, and this was echoed by experts in the GA4GH working group. To address this, we developed a model that deliberately separated the representation of genetic test results from the representation of therapeutic implications. This separation ensured that genetic data could be stored as a stable, semantically consistent record, while therapeutic guidance could be linked dynamically, allowing for future updates or context-specific adaptations. We then mapped this model to HL7 FHIR to test interoperability. For example, a genetic result indicating reduced function in a drug-processing enzyme would be recorded once as a pharmacogenetic test result, while the associated prescribing recommendation for a medicine metabolised by that enzyme would be represented separately as a therapeutic implication in an independently stored knowledge base.

### Final data model

The Delphi-inspired process involved peer reviewers across 10 different countries and included a range of healthcare, laboratory and information technology professionals. Table 1 summarises the geographic and professional spread of reviewers.

**Table 1:** Geographic and professional spread of reviewers involved (N=number of reviewers).

Geography	N	Profession	N
Australia	3	Pharmacist	2
Finland	1	Doctor	5
India	2	Nurse	2
Italy	3	Health IT	13
New Zealand	1	Laboratory	2
Norway	4		
Spain	1		
Sweden	1		
United Kingdom	5		
United States	3		

Through an iterative process across review rounds and editorial meetings we finalised the names of the data elements, their descriptions and intended uses. The data elements that could be bound to existing terminologies, such as HUGO Gene Nomenclature Committee (HGNC) were included, as well as the standardised consensus LOINC and SNOMED CT terms recommended by the Clinical Pharmacogenetics Implementation Consortium (CPIC) [17].

**Fig. 2:**
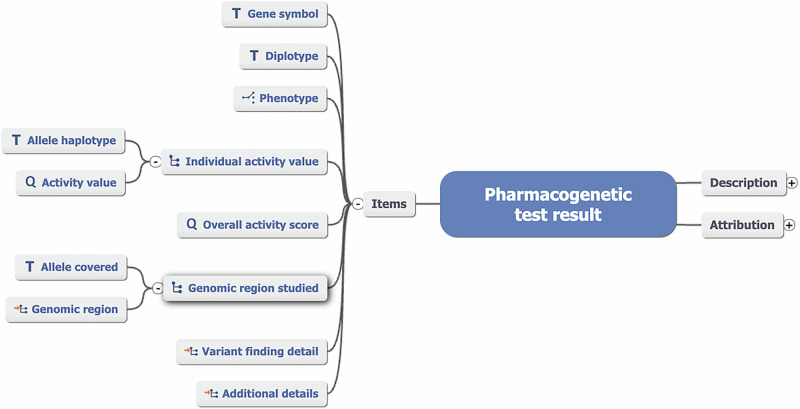
Mind map of the final openEHR data model (archetype), summarising data elements.

The model was structured around a set of discrete data elements designed to capture both the genetic findings and their immediate interpretations, while remaining separate from downstream therapeutic implications. Full details of all the data elements can be found in supplement I and viewed on the CKM website. The approved archetype (v1.0) was published on 22/11/2024 and is visualised as a mind map in Fig. 2.

At its core, the model included standardised identifiers such as the gene symbol, diplotype, and inferred phenotype, which provided the primary link between a patient’s genetic makeup and expected functional consequences. To support more granular representation, the model also incorporated allele haplotype information, together with associated individual activity values and an aggregated overall activity score, enabling consistency across different genes and calculation frameworks.

The model further specified the genomic region studied and the alleles covered, allowing transparency about the scope and limitations of the test. Within this branch, optional details such as variant finding detail and additional contextual information could be recorded, ensuring that the test result could be interrogated or reinterpreted considering future evidence.

Complementary metadata elements, such as description and attribution, ensured that the test was fully documented, supporting provenance, traceability, and quality assurance. Together, these components provided a flexible yet standardised framework for storing pharmacogenetic test results in a way that maintained semantic harmony, accommodated future updates, and supported interoperability across health IT systems.

### Validation and usability testing

Analysis of stakeholder interviews identified several key themes related to the integration, usability, and adoption of the pharmacogenetics data model within NHS systems.

#### Integration into health IT systems

Participants highlighted the importance of aligning the data model with existing NHS digital infrastructure, particularly the Electronic Prescription Service and GP clinical systems. IT experts emphasized the need for interoperability with established data standards such as FHIR and SNOMED CT to ensure seamless data exchange. A key barrier identified was the limited availability of standardized pharmacogenetic decision-support tools within electronic health records (EHRs), which could hinder real-world adoption.

#### Usability and clinical decision-making

General practitioners and a national medical director stressed that for pharmacogenetic data to be actionable, it must be presented in a clear and concise format within existing prescribing workflows. Alerts and clinical decision-support mechanisms were considered essential to minimize cognitive burden and support timely prescribing decisions. Bioinformaticians highlighted the complexity of translating raw genomic data into clinically meaningful outputs and emphasized the need for automated pipelines to process and interpret test results.

#### Implementation and adoption

Stakeholders identified governance, policy, and training as critical factors influencing adoption. Concerns were raised about the regulatory frameworks required to standardise pharmacogenetic data usage across different NHS organizations. Training and awareness-building were seen as essential to ensure confidence in using pharmacogenetic results in prescribing decisions. Participants also emphasized the need for ongoing engagement with policymakers and front-line clinicians to drive implementation.

Overall, the findings reinforced the need for robust technical infrastructure, user-centred design, and clear governance pathways to support the successful deployment of pharmacogenetic data models in NHS clinical practice.

## Discussion

This paper reports the development of an open data model to store pharmacogenetic test results using openEHR and explored requirements for mapping to HL7 FHIR. This adds novel data to the literature on interoperability and standards to support wider use of precision medicine in routine care.

### Implications for clinical practice

This is the first report of an open data model to structure and standardise pharmacogenetic test results for frontline care. Much of the literature still refers to ‘reports’ as the main output for healthcare professionals and patients [18]. We argue that pharmacogenetics at scale requires moving away from detailed reports for specialists towards concise ‘test results’ for generalists, akin to a renal function test guiding dosing decisions. The use of genetic variation in healthcare holds significant potential, but adoption will require innovation that simplifies complexity and meaningfully considers end-users [19].

Pharmacogenetic test results are most valuable within prescribing workflows when integrated with CDS systems [20]. As with other predictive insights, this depends on EHR integration [21]. Reported implementations of pharmacogenetic CDS systems have focussed on individual centres, primarily in the USA, working with their local EHR provider to integrate pharmacogenetics, such as at the Mayo Clinic [22]. The eMERGE-PGx project sought collective implementation across 10 sites and contributed to reports on FHIR use, though publication on outcomes is awaited [23, 24].

One relevant output of eMERGE-PGx was the concept of ‘genomic indicators’, providing a ‘home’ for genomic results within EHRs [25]. Genetic test results differ from existing informatics concepts like ‘observation’ or ‘diagnosis’; fitting them into constructs that do not reflect clinical use may be inappropriate. Individuals may undergo multiple pharmacogenetic tests over a lifetime, requiring decision-making based on cumulative results. This supports a separate concept such as a ‘pharmacogenetic profile’ or ‘genomic indicator’ as the single source of truth.

**Fig. 3:**
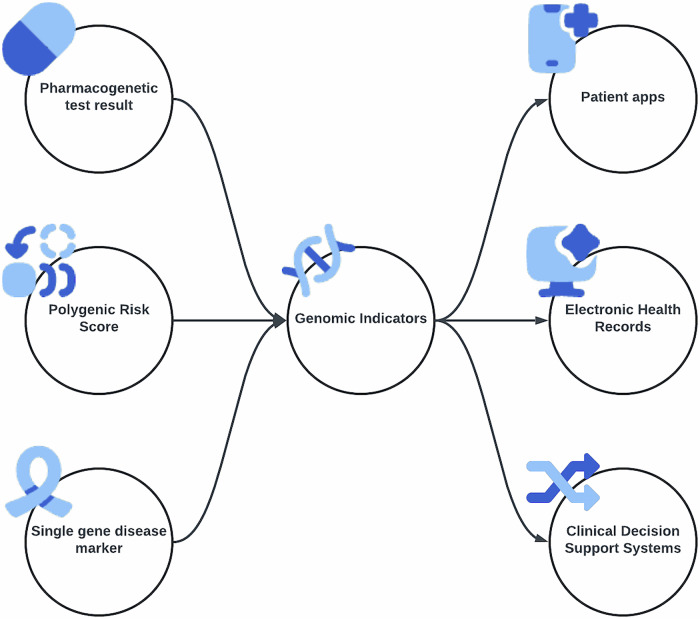
A genomic indicator concept could provide a data model to shift the concept of ‘test reports’ to actionable ‘test results for genomic data that is more widely used across a health system, such as pharmacogenetic test results, polygenic risk scores (e.g. cardiovascular) and single gene disease markers (e.g. inherited breast cancer risk).

The genomic indicator could also extend beyond pharmacogenetics to polygenic risk scores and single-gene disease markers, making results more actionable across health systems rather than static reports. Further work is needed to mature these ideas and develop standards that enable the next layer of innovation (Fig. 3).

### Implications for policy

In the field of health data standards there is continued debate between which standard is most appropriate. For pharmacogenetics, explicit semantic harmonisation was recognised as a critical component for the use of results in EHRs [26]. openEHR offers a collaborative Delphi-inspired methodology and tooling to reach international multi-stakeholder consensus and was therefore a natural place to start [27]. In addition, openEHR promotes a ‘maximum dataset’ philosophy, which means we could gather broad clinical concepts and represent them in the standard. Subsequent mapping to other standards, e.g. FHIR, which takes an 80/20 dataset philosophy is therefore readily feasible, whereas if we started with FHIR, mapping to openEHR (or other standards, such as OMOP) would not have been possible [28]. This approach is increasingly recognised by clinical and digital leaders, with national policies highlighting convergence on openEHR and FHIR to advance interoperability [29, 30].

Successful integration of pharmacogenetics into routine care requires more than technical standards; it also depends on coordinated policy. This should include clinical guidelines for consistent prescribing, professional education to build confidence, and patient and public engagement to foster trust. Policymakers should prioritise adoption of open data standards, while collaboration with professional bodies, such as the Royal College of General Practitioners or the American Medical Association, can embed precision medicine more widely. Finally, regulatory frameworks must safeguard CDS systems as regulated medical devices, ensuring they are assessed for safety and variation. With strong policy, evidence-based innovation, and cross-industry partnerships, pharmacogenetics can move beyond pilots and deliver meaningful benefits at scale.

### Limitations of this study

This study had some limitations. The consensus process involved iterative editorial meetings to reconcile feedback, which may not fully capture all perspectives despite wide international representation. Validation was based on stakeholder interviews rather than live deployment, so further work is needed to explore performance in real-world settings. In addition, while mapping to HL7 FHIR showed feasibility, future studies should assess scalability and adoption across different healthcare environments.

## Conclusion

The integration of pharmacogenetics into routine practice has the potential to significantly improve care and outcomes. However, the lack of open data standards remains a key barrier to widespread implementation. Using openEHR and FHIR, we developed an open data model to represent a pharmacogenetic test result. This enables the structured storage of data and acts as a ‘single source of truth’ to support clinical implementation and CDS systems. This interoperable approach can make pharmacogenetic test results available across healthcare settings and enable precision medicine at scale.

## Supplementary information

Supplement I
