# Supplementary material for: Development and validation of an open data model for pharmacogenetics to enable semantic interoperability in clinical practice
Source: Pharmacogenomics J. 2026 Jun 13;26(3):27. doi: 10.1038/s41397-026-00418-0 (PMC13263137; doi:10.1038/s41397-026-00418-0)
Supplement: Supplementary file 1 — Supplement I [file 41397_2026_418_MOESM1_ESM.pdf]

Pharmacogenetic test result

Header

Concept Name	Pharmacogenetic test result
Concept Description	Findings from pharmacogenetic testing of a single gene, used to predict how an individual may respond to specific medications.
Keywords	pharmacogenetics, laboratory, enzyme, medication, pgx, CPIC, genomics, pharmacogenomics, genotype
Purpose	To record findings from pharmacogenetic testing of a single gene, used to predict how an individual may respond to specific medications.
Use	Use to record findings from pharmacogenetic testing of a single gene, used to predict how an individual may respond to specific medications.<br><br>This archetype has been designed so that one or more instances of this archetype may be nested within the 'Analyte result detail' SLOT in the CLUSTER.laboratory_test_analyte archetype, carried inside the 'Test result' SLOT in the OBSERVATION.laboratory_test_result archetype, to be consistent with the existing approach to laboratory modelling.
Misuse	Not to be used to record information about specific genomic variants. Use one or more of the Genomics variant family of archetypes for this purpose.<br><br>Not to be used to record therapeutic recommendations that are the conclusions or interpretations based on each Pharmacogenetic test result.
References	Caudle KE, Dunnenberger HM, Freimuth RR, Peterson JF, Burlison JD, Whirl-Carrillo M, Scott SA, Rehm HL, Williams MS, Klein TE, Relling MV, Hoffman JM. Standardizing terms for clinical pharmacogenetic test results: consensus terms from the Clinical Pharmacogenetics Implementation Consortium (CPIC). Genet Med. 2017 Feb;19(2):215-223. doi: 10.1038/gim.2016.87. Epub 2016 Jul 21. PMID: 27441996; PMCID: PMC5253119.<br><br>Goar W, Babb L, Chamala S, Cline M, Freimuth RR, Hart RK, Kuzma K, Lee J, Nelson T, Prlić A, Riehle K, Smith A, Stahl K, Yates AD, Rehm HL, Wagner AH. Development and application of a computable genotype model in the GA4GH Variation Representation Specification. Pac Symp Biocomput. 2023;28:383-394. PMID: 36540993; PMCID: PMC9782714.<br><br>Health Level 7 International [Internet]. Genomics Reporting Implementation Guide [cited 2024 Sep 3]. Available from: https://hl7.org/fhir/uv/genomics-reporting/.<br><br>Dolin RH, Boxwala A, Shalaby J. A Pharmacogenomics Clinical Decision Support Service Based on FHIR and CDS Hooks. Methods Inf Med. 2018 Dec;57(S 02):e115-e123. doi: 10.1055/s-0038-1676466. Epub 2019 Jan 3. PMID: 30605914.<br><br>Professional Record Standards Body [Internet]. Using pharmacogenomic information in clinical practice [cited 2024 Sep 3]. Available from: https://theprsb.org/projects/geneticsandmedicines/.<br><br>McDermott JH, Wright S, Sharma V, Newman WG, Payne K, Wilson P. Characterizing pharmacogenetic programs using the consolidated framework for implementation research: A structured scoping review. Front Med (Lausanne). 2022 Aug 18;9:945352. doi: 10.3389/fmed.2022.945352. PMID: 36059837; PMCID: PMC9433561.<br><br>Roosan D, Hwang A, Law AV, Chok J, Roosan MR. The inclusion of health data standards in the implementation of pharmacogenomics systems: a scoping review. Pharmacogenomics. 2020 Nov;21(16):1191-1202. doi: 10.2217/pgs-2020-0066. Epub 2020 Oct 30. PMID: 33124487.

Attribution

Archetype ID	openEHR-EHR-CLUSTER.pharmacogenetic_test_result.v0<br>Original namespace: org.openehr<br>Original publisher: openEHR International<br>Revision: 0.0.1-alpha (in_development)
Other Identification	Build UID: 3b55ac08-5864-40b1-9b46-977680e89461<br>Major Version UID: 0dcffeb4-d693-4de4-878c-bdaa9b93f853<br>Canonical MD5 Hash: D310C8A2534AEFD0FAA0618BD791EDA1

Licencing	Copyright: © openEHR Foundation<br><br>Licence: This work is licensed under the Creative Commons Attribution-ShareAlike 4.0 International License. To view a copy of this license, visit http://creativecommons.org/licenses/by-sa/4.0/.<br><br>IP acknowledgements: This artefact includes content from SNOMED Clinical Terms® (SNOMED CT®) which is copyrighted material of the International Health Terminology Standards Development Organisation (IHTSDO). Where an implementation of this artefact makes use of SNOMED CT content, the implementer must have the appropriate SNOMED CT Affiliate license - for more information contact https://www.snomed.org/snomed-ct/get-snomed or info@snomed.org.
Original Author/Publisher	Author name: Ian McNicoll<br>Organisation: freshEHR Clinical Informatics<br>Email: ian@freshEHR.com<br>Date originally authored: 2023-05-22
Current Custodian	Custodian Organisation: Apperta UK<br>Custodian Namespace: uk.org.clinicalmodels<br>Current contact: Ian McNicoll (ian@freshehr.com>
Other Contributors	John McDermott, University of Manchester<br>Videha Sharma, University of Manchester<br>William Newman, University of Manchester<br>Jessica Keen, North West Genomic Medicine Service Alliance

Items

Gene symbol<br>Text<br>Mandatory	The official gene symbol approved by the HGNC, which is a short abbreviated form of the gene name.<br>*Comment: Coding with an external terminology is preferred, where possible. Preferably this should be coded with terms from the HGNC (HUGO Gene Nomenclature Committee) nomenclature, using 'http://www.genenames.org' as the terminology identifier. For example: 'http://www.genenames.org::CYP2C9 | CYP2C9 |'.*	
Diplotype<br>Text<br>Optional	A representation of the diplotype as a coded term or text string, commonly including the gene symbol, gene change descriptors or star allele diplotypes.<br>*Comment: Currently there is no definitive standardised method of representing all diplotypes.*	
Phenotype<br>Text<br>Optional, repeating	The estimated pharmacological impact of the identified genotype.<br>*Comment: Phenotype values should be based on the CPIC recommendations (see: https://www.ncbi.nlm.nih.gov/pmc/articles/PMC5253119/), preferably using the recommended SNOMED CT or LOINC terms. The free text choice with multiple occurrences permits use of local text/coded text value sets that can be split into different categories (e.g. functional status, metabolising speed, transporter function), as required.*	
Individual activity value<br>Cluster<br>Optional, repeating	A list of enzyme activity values each associated with a specific allele, which are used to derive the Overall activity score.	
Allele haplotype<br>Text<br>Optional	The allele haplotype descriptor associated with the activity value.<br>*Comment: For example: '*36'.*	
Activity value	An associated, derived activity value, based on known	Property: Qualified real

Activity value<br>Quantity<br>Optional	An associated, derived activity value, based on known association with specific genotypes, which is required to assess metaboliser status for some phenotypes.<br>*Comment: For example: '0.5'.*	Property: Qualified real<br>Units: 0.0..10.0
Overall activity score<br>Quantity<br>Optional	The overall activity score for this test result, derived from the total of each allele activity value.<br>*Comment: For example: '1.5'.*	Property: Qualified real<br>Units: 0.0..10.0
Genomic region studied<br>Cluster<br>Optional	A list of the genomic region(s) studied by this pharmacogenetic test. Typically, this would be a list of SNP rsNumbers but other variant or genomic region identifiers can be used.<br>*Comment: This element is significant to interpretation of the test, as individual pharmacogenetic tests may differ in their coverage of significant alleles/variants.*	
Allele covered<br>Text<br>Optional, repeating	The haplotype description of an allele covered by the test.<br>*Comment: In pharmacogenetic test results, allele coverage is commonly described as a list of alleles, usually as 'star alleles'. For example: '*3'.*	
Genomic region<br>SLOT (Cluster)<br>Optional, repeating	An identifier or descriptor of a genomic region studied, typically an allele, but also a variant or other genomic region.<br>*Comment: It is recommended to use the openEHR-EHR-CLUSTER.knowledge_base_reference.v1 archetype to carry the SNP rsNumber or other genomic region identifier. For example: Knowledge base name: 'http://terminology.hl7.org/NamingSystem/v3-dbSNP'. Item name: 'rs3918290'.*	**Include:**<br>openEHR-EHR-CLUSTER.knowledge_base_reference.v1
Variant finding detail<br>SLOT (Cluster)<br>Optional, repeating	Details of a single variant finding.<br>*Comment: It is recommended to use the openEHR-EHR-CLUSTER.genomic_variant_result.v1 archetype as the main container for detailed variant information, possibly making use of other structured variant archetypes or as an attached file in a recognised standard format, such as the GA4GH VRS format.*	**Include:**<br>openEHR-EHR-CLUSTER.genomic_variant_result.v1
Additional details<br>SLOT (Cluster)<br>Optional, repeating	Structured details or questions about the pharmacogenetic test result.	**Include:**<br>All not explicitly excluded archetypes
